# Knowledge and use of emergency contraception among women in the Western Cape province of South Africa: a cross-sectional study

**DOI:** 10.1186/1472-6874-7-14

**Published:** 2007-09-12

**Authors:** Landon Myer, Regina Mlobeli, Di Cooper, Jennifer Smit, Chelsea Morroni

**Affiliations:** 1 Infectious Diseases Epidemiology Unit, School of Public Health & Family Medicine, University of Cape Town, Cape Town, South Africa; 2 Department of Epidemiology, Mailman School of Public Health, Columbia University, New York, USA; 3 Women's Health Research Unit, School of Public Health & Family Medicine, University of Cape Town, Cape Town, South Africa; 4 Reproductive Health and HIV Research Unit, Department of Obstetrics and Gynaecology, University of the Witwatersrand, Durban, South Africa

## Abstract

**Background:**

Emergency contraception (EC) is widely available free of charge at public sector clinics in South Africa. At the same time, rates of teenage and unintended pregnancy in South Africa remain high, and there are few data on knowledge of EC in the general population in South Africa, as in other resource-limited settings.

**Methods:**

We conducted a cross-sectional, interviewer-administered survey among 831 sexually active women at 26 randomly selected public sector clinics in the Western Cape province.

**Results:**

Overall, 30% of the women had ever heard of EC when asked directly, after the method was described to them. Only 15% mentioned EC by name or description spontaneously. Knowledge of EC was independently associated with higher education, being married, and living in an urban setting. Four percent of women had ever used EC.

**Discussion:**

These data suggest that knowledge of EC in this setting is more common among women of higher socioeconomic status living in urban areas. For EC to play a role in decreasing unintended pregnancy in South Africa, specific interventions are necessary to increase knowledge of the method, where to get it, and the appropriate time interval for its use before the need for EC arises. Future health promotion campaigns should target rural and low socioeconomic status communities.

## Background

Expanding the number of family planning options available to women is a critical part of increasing contraceptive coverage, decreasing unintended pregnancies and reducing maternal morbidity and mortality around the globe [1,2]. Hormonal emergency contraception (EC) is an important contraceptive option in both developed and developing country settings. EC is the only form of hormonal contraception that can reduce the risk of pregnancy after unprotected intercourse or when a planned contraceptive method fails; increasing the availability and promotion of EC has the potential to reduce the incidence of unintended pregnancies if used when the need arises and in turn reduce associated social and healthcare costs [3,4]. Data from several settings have shown that increasing hormonal EC knowledge and access does not increase levels of unprotected intercourse [5].

Although several contraceptive methods, including EC, are available and free to users at all public sector health facilities across the country, high rates of teenage and unintended pregnancy in South Africa persist. It is estimated that up to 75% of pregnancies in South Africa are unintended, with the highest proportion among adolescents [6-8], and the incidence of sexual assault is also very high [9].

EC is provided free of charge at public sector health facilities in South Africa, usually in the form of "cut-up" regular combined oral contraceptives (COCs). EC products, either COCs or a dedicated levonorgestrel-only product, are also available "behind the counter" at many private pharmacies. However, the price of dedicated EC products sold in pharmacies is high and may not be affordable to many. In order to realise the public health benefits of widespread hormonal EC availability, potential users must be well informed about EC. Specifically, women must know that EC exists, know the time limits within which it may be effective, and know that it can be obtained from public clinics [10]. Without this knowledge, women will miss the opportunity to access free EC at public sector health facilities, where 84% of contraceptive users in South Africa obtain their contraception [6]. Given the importance of EC-related knowledge, we examined levels of awareness and uptake of hormonal EC among women attending public sector primary level clinics in the Western Cape Province of South Africa.

## Methods

We conducted a cross-sectional survey at 26 public sector primary care clinics in one urban (17 clinics around Cape Town) and one rural (9 clinics in the Boland/Overberg region) health region of the Western Cape Province, South Africa, from November 2004 to February 2005. The Western Cape is home to approximately 5 million people, the majority living in or around Cape Town. Reproductive health services are relatively well developed in this setting, with a contraceptive coverage rate of 74% among sexually active women ages 15–49. The urban region is characterised by a large metropolitan area (the only major city in the province) with densely populated residential areas where clinics are located; the rural region (one of four in the province) is characterised by a commercial agricultural economy interspersed with small towns, each of which has a primary care clinic. In each region, a random sample of clinics was selected with probability of selection weighted by patient load based on clinic usage statistics, following the sampling strategy of a previous survey of EC conducted in 1999/2000 [11].

At each clinic over a two-day period, trained female interviewers with a minimum of high school education approached consecutive women exiting the clinic to participate. Women were eligible if they had ever had sexual intercourse and were between the ages of 15 and 49. The number of women interviewed at each facility was proportional to clinic size and varied from 11 to 52. Women were approached to participate regardless of their reason for being at the clinic and refusals were minimal (<2%). Semi-structured interviews were conducted in a private room on-site in participants' preferred language and lasted approximately 15 minutes. The interviews employed standard questionnaire items that we have used for research on EC and women's reproductive health in this setting [11], without distinction between hormonal and non-hormonal EC methods, and included pilot testing and standardized phrasing for administration in different local languages.

Stata version 8.0 (Stata Corporation, College Station, Texas, USA) was used for statistical analyses. Chi-square, Fisher's exact, and student's t-tests were used to determine factors associated with awareness of EC. Logistic regression models using generalized estimating equations with robust variance estimators were used to examine the predictors of awareness of EC, taking into account the clustered sampling of participants within clinics [12]. Independent variables were entered into the model if they demonstrated an appreciable crude association (p < 0.10) with the outcome, and were retained if their association persisted, or if their removal altered associations involving other covariates. The results of this model are presented as adjusted odds ratios (OR) and 95% confidence intervals (CI).

All participants provided informed consent, and approval to conduct the survey was granted by the Provincial Department of Health and the Research Ethics Committee of the University of Cape Town.

## Results

### Socio-demographic characteristics

Of the 831 sexually active women who participated in the study, 624 (75%) were from the urban health region and 207 (25%) from the rural health region (Table 1). Most participants were attending the clinic on the day of the interview for either specific medical problems (34%, n = 283) or for postnatal care (30%, n = 249); 19% of the sample (n = 158) were at the clinic for family planning services specifically. The median age was 26 years (IQR, 21–34) and the median level of education was grade 10 (equivalent to 10 years of formal schooling). Most participants spoke either Afrikaans or Xhosa as their home language (47%, n = 391 and 44%, n = 366, respectively). Eighty-two percent of participants (n = 688) had been pregnant at least once; for 61% of the participants who had ever been pregnant (n = 420), their last pregnancy was unintended.

**Table 1:** Description of the study sample, overall and by study region, of sexually active women attending public sector clinics in the Western Cape Province, South Africa; all cells are N (%) unless otherwise specified

*Characteristic*	*Urban Region N = 624*	*Rural Region N = 207*	*p-value*^1^	*Total N = 831*
Median age	28	25	0.069	26
Home language				
Xhosa	275 (44)	106 (51)		381 (46)
Afrikaans	283 (45)	100 (48)		383 (46)
English/Other	65 (11)	1 (<1)		66 (8)
Median years of schooling	10	10	0.890	10
Marital status: Married	237 (39)	68 (33)	0.143	305 (37)
Last pregnancy unintended ^2^	300 (59)	120 (70)	0.008	420 (61)
Method used for pregnancy prevention at last sexual intercourse ^3^				
No method	160 (26)	68 (33)	0.056	228 (28)
Oral contraceptive	49 (8)	8 (4)	0.045	57 (7)
Injectable	272 (44)	100 (48)	0.274	372 (45)
Male condom	132 (21)	36 (17)	0.220	168 (20)
Female sterilization	67 (11)	13 (6)	0.058	80 (10)

1. P-values are calculated using chi-square tests (for proportions) and Wilcoxon rank-sum tests (for medians)

2. Percentages are of women who report at least one past pregnancy.

3. Participants could report using more than one method.

Just under three-quarters of women (72%, n = 598) were using a clinic-supplied method of contraception at last sexual intercourse. The most commonly used methods at last sexual intercourse were injectables (45%, n = 269), followed by the male condom (20%, n = 120) and female sterilization (9%, n = 55). Twenty-one percent of participants (n = 175) were protected from both pregnancy and sexually transmitted infections (STI) the last time they had sexual intercourse, with 10% using a condom and an effective non-barrier contraceptive (n = 83), and 11% using a condom alone (n = 91). Fifty-one percent (n = 424) were protected only from pregnancy through the use of non-barrier contraception. Twenty-nine percent of women (n = 241) were not using any method. Of the 241 women not using any method, 17% (n = 50) were attending the clinic for post-natal care, while 42% (n = 101) were at the clinic for specific medical problems, and 10% (n = 24) were attending family planning services on the day of the interview.

### Awareness of emergency contraception

Fifteen percent of women (n = 126) mentioned EC by name or description spontaneously when asked if there was anything a woman could do soon after unprotected sexual intercourse to try to prevent pregnancy (111/750 women who were not sterilised, 15%) (Table 2). The percentage of women who spontaneously mentioned EC in the rural region (7%, n = 15) was lower than in the urban region (18%, n = 111) (p < 0.001). Overall, 30% of the women in the entire sample (n = 253) had ever heard of EC when asked directly, after the method was described to them (including 230/750 women who were not sterilised, 31%). The level of EC awareness was particularly low in the rural region with only 17% of women (n = 34) being aware of EC on direct questioning compared with 35% of women in the urban region (n = 219).

**Table 2:** Awareness, knowledge and use of emergency contraception among sexually active women attending public sector clinics in the Western Cape Province, South Africa; all cells are N (%)

*Characteristic*	*Urban Region N = 624*	*Rural Region N = 207*	*Total N = 831*
Spontaneously mentioned EC as something that can be done after unprotected sex to try to prevent pregnancy	111 (18)	15 (7)	126 (15)
Heard of EC when asked directly	219 (35)	34 (17)	253 (30)
*Among those who have heard of EC*			
Did not know if EC available at public clinics	79 (36)	14 (41)	93 (37)
Thought EC were not available at public clinics	19 (9)	2 (6)	21 (8)
Ever been told about EC by health care provider	53 (24)	11 (32)	64 (25)
Did not know about time interval for use	160 (73)	30 (88)	190 (75)
Ever used EC	31 (14.0)	3 (9)	34 (13)
Ever used EC, total sample	31 (5)	3 (1)	34 (4)
*Among those who have ever used EC*			
*Number of times used EC*			
Once	23 (74)	3 (100)	26 (76)
More than once	8 (26)	0	8 (24)
*Source of EC at last use*			
Public clinic	0	1 (33.3)	1 (3)
Private doctor/clinic	15 (48)	1 (33.3)	16 (47)
Pharmacy	14 (45)	1 (33.3)	15 (44)
Hospital or district surgeon for rape	2 (6)	0	2 (6)

Respondents who were aware of EC most commonly reported that they had first heard about EC from friends or family members (40%, n = 100) or at the clinic (27%, n = 69). Other sources of information about EC were the mass media (9%, n = 23) and school (5%, n = 13). Thirty-seven percent of subjects (n = 93) who had heard of EC did not know whether they could obtain EC from the clinic they were visiting, and 8% (n = 21) believed they could not get it at that clinic (Table 2).

In addition, most participants (75%, n = 190) who were aware of EC did not know about the appropriate interval for efficacy between unprotected sex and taking EC (Table 1) (based on recent evidence we considered this interval to be up to 72–96 hours after sexual intercourse) [13]. Approximately 41% of participants (n = 104) said they did not know that there was a time interval for use; 24% (n = 61) believed that it could be taken only immediately after, the morning after, or within 12 or 24 hours of intercourse. Nine percent (n = 23) reported that EC must be taken within two days, and 1% (n = 2) thought it could be taken up to one week after intercourse. Of those who were aware of EC, only 26% (n = 64) had ever been told about EC by a health care provider.

Unadjusted associations between participant characteristics and awareness of EC are shown in Table 3. Women over the age of 20 were significantly more likely to have heard of EC than teenagers or women 40 or older. More educated women and women whose most recent pregnancy was not unintended were also more likely to have heard of EC. Knowledge of EC was also associated with the type of method used at last sexual intercourse: women using the oral contraceptive pill were more likely to have heard of EC than other women. Knowledge of EC was lowest among women who used a condom alone for pregnancy prevention at last sexual intercourse. The strongest independent predictors of EC awareness were location in an urban area (OR: 2.1; 95% CI: 1.3–3.4) and having a higher level of education (OR: 5.9; 95% CI: 3.3–10.5) (Table 4). However, the crude associations involving participant age, unintended pregnancy and contraceptive methods used did not persist in the multivariate model. These results were not altered when the model was restricted to women who had not been sterilized (data not shown).

**Table 3:** Unadjusted (crude) associations with awareness of emergency contraception among sexually active women attending public sector clinics in the Western Cape Province, South Africa (n = 831)

*Characteristic, %*	*Heard of EC n = 252*	*Not heard of EC n = 579*	*p-value*^1^
Total	30.4	69.6	
Region			
Urban	34.9	65.1	<0.001
Rural	16.5	83.5	
Age (years)			
15–19	23.1	76.9	0.009
20–39	34.2	65.8	
40–49	27.4	72.6	
Education			
Less than high school (None/primary)	11.6	88.4	<0.001
Some high school or greater	33.4	66.6	
Marital status			
Married	33.8	60.2	<0.001
Unmarried	25.3	74.7	
Main language spoken			
Xhosa	12.1	87.9	<0.001
Afrikaans	40.6	59.4	
English	80.6	19.4	
Other	50.0	50.0	
Last pregnancy unintended			
Yes	29.0	71.0	0.020
No	37.3	62.7	
Method used for pregnancy prevention at last sexual intercourse			
No method	30.1	69.9	0.013
Oral contraceptive	56.0	44.0	
Injectable	30.6	69.4	
Male condom	24.7	75.3	
Female sterilization	29.9	70.1	

1. P-values are calculated using chi-square and Fisher's exact tests, as appropriate

**Table 4:** Results of multivariate modelling to determine factors independently associated with awareness of EC among sexually active women attending public sector clinics in the Western Cape Province, South Africa (n = 831) ^1^

*Characteristic*	*Odds ratio*	*95% CI*
Region		
Urban	2.1	1.3–3.4
Rural	1.0 (Ref)	
Education		
Some high school/greater	5.9	3.3–10.5
Less than high school (None/primary)	1.0 (Ref)	
Marital status		
Married	1.4	1.0–2.1
Not married	1.0 (Ref)	
Main language spoken		
Xhosa	0.04	0.02–0.08
Afrikaans	0.3	0.2–0.6
English	1.0 (Ref)	

1. Variables examined in the model examined during the model building process, but not included in the final model were: age, unintended pregnancy status, and method use at last sexual intercourse (see methods section for description of model-building strategy).

### Use of emergency contraception

Thirty-four women had ever used EC, 13% of the 253 who were aware of EC and 4% of the total sample of 831 women (and 4% of unsterilized women); most of these had only ever used it once (76%, n = 26). The main sources of supply were private doctors and pharmacies (Table 2).

## Discussion

The findings of this study indicate that awareness of EC is relatively low among women both in the rural and urban region of the Western Cape province, South Africa. Overall, awareness is lower in South Africa than recent data from Europe and North America [14], but is similar to or higher than what has been reported in other developing countries including Mexico, India, Kenya and Nigeria [15-18]. A South African survey of EC knowledge and use, which was conducted in 1999/2000 found that nationally 23% of women knew of EC; the Western Cape provincial figures were 34% for the urban region and 18% for the rural region [11]. The current study, which employed the same methodology and sampling, found no substantial change in these statistics over this 6-year period.

We found that the lowest level of EC awareness was among teenagers, which conflicts with other data from South Africa and internationally [11,14]. In light of the HIV epidemic in South Africa, increasing emphasis is being placed on the promotion of condom use, especially among teenagers, for both STI and pregnancy protection [19]. Because EC is advocated as a back-up contraceptive method for condom failure or non-use, it was concerning to find that EC awareness was lowest among young women and women using a condom alone at last sexual intercourse. Women with a higher level of education and women attending urban clinics had the greatest levels of EC awareness. Women with more education and who live in urban areas may get reproductive health information from sources other than public health clinics. In fact, we found that a smaller proportion of women who were aware of EC had been told about EC by a health care provider in the urban area (24%) than in the rural area (33%). Educated women may also have a greater incentive to obtain information on strategies to delay childbearing, and thus seek out information for themselves about options such as EC [20,21].

These data suggest that most women who had heard about EC did so from friends and family. Given that information about EC is relatively simple to convey accurately, and in light of this finding, peer education approaches may be useful in increasing EC awareness. More generally these findings indicate a substantial unmet need among women for information on EC and a need for greater client-health care provider dialogue regarding EC, including the existence of EC, its availability at public sector clinics, and the timeframes involved in its use after unprotected sex. Relaying basic information on EC needs to become part of routine reproductive health counselling and specific health service interventions to improve EC awareness need to be designed, implemented and evaluated in South African and other resource-limited settings.

Accurate EC knowledge was lacking, even among those who were aware of the method. Without accurate knowledge of EC it is unlikely that those who need it will be able to access it within the appropriate window of opportunity [10]. Despite the availability of EC free of charge at all public sector primary level clinics in South Africa for several years, and the strong promotion of EC in the South African national contraception policy guidelines [22], very few women in this study had ever used it. This very low level of EC use is occuring in the context of high levels of unprotected intercourse and unintended pregnancy in this study population.

Almost all of the women who had used EC had purchased it from private sources (doctors, clinics or pharmacies), suggesting a lack of awareness of EC availability at public clinics. This may also reflect the fact that women who know and use EC are of higher socioeconomic status than women who do not. Most women either did not know if EC was available at the clinic they were attending, or thought that it was not available at the clinic. Pharmacies have less restricted operating hours and are open on weekends and after hours, which could explain the higher uptake of EC, despite the costs involved. Making EC more affordable through pharmacies or other similar outlets may enhance the provision of information and access in addition to improving public sector information and distribution.

Our study has several limitations. First, this was a relatively small survey conducted in one part of South Africa. The results require further investigation in other settings in Sub-Saharan Africa, particularly where reproductive health knowledge and health care infrastructure may be more limited. Additionally, this survey was conducted among individuals attending public health clinics, who are likely to have higher health-related knowledge than women from a general population sample. Related to this our study focused on the Western Cape Province, which has a better reproductive health infrastructure than most other areas of South Africa, and a result awareness and uptake of EC may be higher in this sample compared to other parts of the country.

## Conclusion

Given the important role that health service providers play in women's knowledge and perceptions of contraceptive methods [23], future research needs to be conducted in South Africa and other settings among healthcare providers themselves. For EC to play a role in increasing contraceptive coverage and decreasing unintended pregnancy in South Africa, specific health service interventions are necessary so that all South African women know about the method, where to get it, and the appropriate time interval for its use before the need for EC arises.

## Competing interests

The authors declare that they have no competing interests.

## Authors' contributions

LM participated in the design of the study, data analysis, and drafted the manuscript. RM participated in the design and conduct of the study. DC participated in the design of the study. JS participated in the design of the study. CM participated in the design and conduct of the study, data analysis, and drafted the manuscript. All authors read and approved the final manuscript.

## Pre-publication history

The pre-publication history for this paper can be accessed here:


